# Molecular cloning, expression, and purification, along with *in silico* epitope analysis of recombinant enolase proteins (a potential vaccine candidate) from *Candida albicans* and *Candida auris*

**DOI:** 10.3389/ffunb.2024.1399546

**Published:** 2024-05-31

**Authors:** Manisha Shukla, Rohit Singh, Pankaj Chandley, Soma Rohatgi

**Affiliations:** ^1^ Department of Biosciences and Bioengineering, Indian Institute of Technology (IIT), Roorkee, Roorkee, India; ^2^ Department of Biotechnology, Pandit S.N. Shukla University, Shahdol, India

**Keywords:** *Candida auris*, systemic candidiasis, vaccine candidate, enolase, epitopes, *Candida albicans*

## Abstract

*Candida albicans* is the predominant cause of systemic candidiasis, although other non albicans Candida species are progressively becoming more widespread nowadays. *Candida auris* has emerged as a deadly multidrug-resistant fungal pathogen, posing a significant threat to global public health. In the absence of effective antifungal therapies, the development of a vaccine against *C. auris* infections is imperative. Enolase, a key glycolytic enzyme, has emerged as a promising vaccine candidate due to its immunogenic properties and essential role in fungal virulence. Herein, full-length Enolase gene sequences from *C. albicans* and *C. auris* were cloned into suitable expression vector and transformed into *Escherichia coli* expression hosts. Recombinant Enolase proteins were successfully expressed and purified using affinity chromatography under native conditions, followed by SDS-PAGE characterization and Western blot analysis. CD spectroscopy verified the existence of expressed proteins in soluble native conformation. Preliminary in silico studies verified the immunogenicity of recombinant Enolase proteins isolated from both *C. albicans* and *C. auris*. Furthermore, bioinformatics analysis revealed conserved B-cell and T-cell epitopes across *C. albicans* and *C. auris* Enolase proteins, suggesting potential cross-reactivity and broad-spectrum vaccine efficacy. Our findings are anticipated to play a role in advancing therapeutic as well as diagnostic strategies against systemic candidiasis.

## Introduction

Systemic candidiasis stands out as a prevalent nosocomial bloodstream infection on a global scale, affecting patients in intensive care units (ICUs) (Warnock, 2007; Soriano et al., 2023). *Candida albicans* has long been recognized as a predominant pathogen responsible for candidiasis. However, the emergence of non-albicans *Candida* (NAC) species, including *C. auris*, contributes to approximately 65% of *Candida* infections (Papon et al., 2013; Whibley and Gaffen, 2015). It has been estimated that over 250,000 individuals globally are affected by systemic candidiasis each year, which results in about 50,000 fatalities (Pappas et al., 2018). According to the Centers for Disease Control and Prevention (CDC), the global mortality rate for systemic candidiasis ranges from 30–70 percent, despite treatment with antifungal medications (Calandra et al., 2016). The emergence of antifungal drug resistance, coupled with high mortality rates and the increasing prevalence of infections caused by NAC species, especially multidrug resistant *C. auris*, has drawn urgent need for alternative immunotherapy approaches for the treatment of systemic candidiasis. Currently, there are no approved immunotherapeutic strategies available for the treatment of systemic candidiasis. Despite the identification of numerous anti-*Candida* vaccine candidates, only a small subset has undergone clinical trial evaluation thus far. Strategies for anti-*Candida* vaccines have included the investigation of many live attenuated strains, recombinant proteins, fungal cell wall polysaccharides, and/or glycoconjugates (Wan Tso et al., 2018). Among the leading vaccine candidates for systemic candidiasis, Enolase protein stands out as a frontrunner.

The Enolase 1 gene, also known as 2-phospho-D-glycerate hydrolase, belonging to the Enolase superfamily, has emerged as one of the leading vaccine candidates due to its well-established role in fungal virulence (Ko et al., 2013). This enzyme is highly expressed in the cytosol and plays a role in the glycolytic pathway, additionally showing transglutaminase activity (Reyna-Beltrán et al., 2018). Besides its role in glycolysis, Enolase has been implicated in various immunological processes and is considered a virulence factor (Lim et al., 2021). In addition to its cytosolic localization, it is secreted into the extracellular medium (He et al., 2022), released into extracellular vesicles (Karkowska-Kuleta et al., 2020), and is also anchored on the fungal cell surface (Satala et al., 2020a). It has been seen that *Candida* cell surface attachment of Enolase is mediated through Als3-binding (Karkowska-Kuleta et al., 2021), and cell wall Enolase further helps in fungal adhesion to various medical devices (Núñez-Beltrán et al., 2017). Exposed enolase on the surface of *C. albicans* cells has been reported to participate in binding human plasminogen, leading to increased invasion of human brain microvascular endothelial cells (Jong et al., 2003; Funk et al., 2016). Numerous research investigations have linked Enolase to its involvement in facilitating microbial attachment to host extracellular matrix proteins such as fibronectin, laminin, and vitronectin (Satala et al., 2020b). It plays a role in facilitating fungal adhesion to human tissues, activating fibrinolysis, and degrading the extracellular matrix (Jong et al., 2003; Silva et al., 2014).

Sundstorm et al., reported that Enolase acts as a prominent humoral immunogen, stimulating both humoral and cell-mediated responses in mice (Sundstrom et al., 1994). In mice, the passive transfer of antibodies against Enolase was seen to offer partial protection against systemic candidiasis (Van Deventer et al., 1996). Another study highlighted the immunogenicity of recombinant Enolase from *C. albicans* and its ability to provide protection against systemic candidiasis by reducing fungal burden and increasing survival in Enolase-immunized mice (Montagnoli et al., 2004). Along with increase in immunogenicity, the protection observed in Enolase vaccinated mice was attributed to induction of a Th1 mediated cellular immune response (Montagnoli et al., 2004). Li et al., demonstrated that immunization with purified recombinant Enolase from *C. albicans* conferred significant protection against tissue damage following challenge with two different strains of *C. albicans*. Protection in mice was associated with a reduced fungal burden, increased titers of Enolase-specific antibodies (IgG1 and IgG2a), increased secretion of Th1 (IL-12 and IL-8) and Th2 (IL-10) cytokines, and effective killing of phagocytosed yeasts by neutrophils (Li et al., 2011). Notably, oral immunization using *Lactobacillus casei* displaying Enolase 1 from *C. albicans* elicited a strong IgG response and increased the survival rate of the vaccinated mice against systemic candidiasis (Shibasaki et al., 2014). Recently, Leu et al., demonstrated improved survival rates, decreased fungal loads, and lowered levels of inflammatory cytokines in mice treated with a single-chain variable fragment monoclonal antibody (CaS1) targeting recombinant *C. albicans* Enolase during candidiasis (Leu et al., 2020).

The aim of this study was to identify and isolate the Enolase gene from *C. auris*, a multi drug resistant (MDR) strain, circulating in the Indian subcontinent, and compare it with the Enolase gene of *C. albicans*, as a reference strain. This study presents the molecular cloning followed by expression and purification of recombinant Enolase proteins of *C. albicans* (SC3514) and *C. auris* (South Asian clade) strains in a prokaryotic expression system. The recombinant Eno-albicans and Eno-auris proteins were purified under native soluble conditions and subsequently dialyzed for further analysis. Circular dichroism (CD) spectroscopy was performed to confirm the secondary structure of the Enolase proteins. Further, the B-cell and T-cell epitopes belonging to Eno-albicans and Eno-auris proteins were predicted using in silico immunoinformatics approaches, to compare their immunogenic and vaccine potential. Our results show that there is a high degree of homology between Enolase proteins belonging to *C. albicans* and *C. auris* species, and they can be used for the development of therapeutic as well as diagnostic strategies against systemic candidiasis.

## Materials and methods

### Chemicals and strains

*C. albicans* strain SC5314 (sourced from Prof. R. Prasad, Amity University, Gurgaon, India) and *C. auris* South Asian strain (Clade 1 CBS 470280) (sourced from Prof. M R Shivaprakash, PGIMER Chandigarh, India) were used for this work. The pQE30Xa vector (Qiagen, Germany) having an N-terminus 6-histidine tag was used for cloning of Enolase protein followed by its expression. Bacterial cultures were done using Luria-Bertani (LB) broth and agar supplemented with Ampicillin (100 μg/ml) and Kanamycin (25 μg/ml) antibiotics. Restriction enzymes used for cloning and amplification were obtained from NEB. The cloning and expression of both recombinant Enolase proteins were performed in *E. coli* XL1-blue and *E. coli* SG13009 strains, respectively. Ni-NTA affinity matrix for protein purification was procured from Qiagen. Sabouraud’s Dextrose broth (SAB) and agar media from HiMedia were utilized for routine *Candida* cultures.

### Culture conditions

All fungal strains were stored at -80°C in SAB with 15% (vol/vol) glycerol. Glycerol stocks of *C. albicans* and *C. auris* strains were utilized for inoculation and subsequently cultured at 30°C and 37°C, respectively, in SAB broth for 48 hours with continuous shaking at 180 rpm (Chandley et al., 2022).

### Cloning of recombinant enolase genes from *C. albicans* and *C. auris*

Genomic DNA was isolated from *C. albicans* and *C. auris* strains using the QIAamp DNA Mini Kit (Qiagen), according to manufacturer’s protocol. PCR amplification of Enolase gene (1323 bp, 440 aa) of *C. albicans* strain SC5314 was performed using gene-specific primers (**Table 1**) and Phusion DNA polymerase (NEB) (Shukla et al., 2021a). The reaction mixture, totaling 25 µL, comprised 5X GC Buffer, 0.2 µM of forward and reverse primers, 200 µM dNTP mix, 50 ng of *C. albicans* genomic DNA, and 2.5 Units of Phusion DNA polymerase. PCR reactions were conducted using the GeneAmp 2700 PCR system. PCR cycling involved an initial denaturation at 98°C for 2 minutes, followed by 30 cycles of denaturation at 98°C for 30 seconds, annealing at 57°C for 1 minute, and extension at 72°C for 1.5 minutes. Final extension was performed at 72°C for 10 minutes.

**Table 1:** Primers and restriction enzymes used for PCR amplification and expression cloning of Enolase fragments from *C. albicans and C. auris*.

Enolase	Amplicons	Primer Sequence with Restriction Enzyme (5’-3’)	Base pairs	Amino acids^a^
Enolase *C. albicans*	Eno alb. FP	CCCCCGGATCCATGTCTTACGCCACTAAAATCC	1323 bp	440 aa
	Eno alb. RP	CCCCCCTGCAGCAATTGAGAAGCCTTTTGGAAAT		
Enolase *C. auris*	Eno aur. FP	CCCCCGGATCCATGGCCGTCACTAAAATTCATG	1320 bp	439 aa
	Eno aur.RP	CCCCCCTGCAGTTACAAGTTTTGAGCAGCCTTG		

a Besides the corresponding amino acids for Enolase fragments, the constructs included additional 31 aa from vector.

PCR amplification of Enolase gene (1320 bp, 439 aa) of *C. auris* strain (Clade 1 CBS 470280) was performed using gene-specific primers (**Table 1**) and Ex-Taq DNA polymerase (Takara Bio). The similar PCR reaction including temperature cycling parameters was employed as those used for amplifying Enolase of *C. albicans*, except that instead of 5X GC Buffer and Phusion polymerase, 10X Ex-Taq buffer and 2.5 Units of Ex-Taq DNA polymerase was used. The Enolase fragments were purified and cloned into corresponding restriction sites of *E. coli* expression vector pQE30Xa (Qiagen). Transformants were screened on LB ampicillin plates and positive clones were verified through restriction digestion and confirmed by sequencing.

### Induction check of recombinant enolase proteins from *C. albicans* and *C. auris*

For protein induction and expression check, the Enolase gene constructs belonging to *C. albicans* and *C. auris* were re-transformed in *E. coli* strain SG13009 (Qiagen). The colonies obtained were proceeded with small scale bacterial cultures. Overnight grown primary cultures (1 ml) at 37°C and 220 rpm were used to inoculate secondary cultures (5 ml) the next day. The bacterial cultures were allowed to grow for 3 hours at 37°C and 220 rpm in orbital shaker. After the OD_600_ was obtained in the range of 0.4 - 0.6, protein expression was induced by adding 1.0 mM isopropyl-β-D-thiogalactopyranoside (IPTG) and kept for 8 hours at 18°C and 220 rpm in orbital shaker. After 8 hours, the induced cultures were centrifuged along with un-induced controls, and the pellets obtained were stored at -20°C. The un-induced and induced pellets were dissolved in SDS loading dye, heat-denatured and analyzed on SDS PAGE.

### Expression and purification of recombinant enolase proteins from *C. albicans* and *C. auris*

The recombinant Enolase proteins from *C. albicans* and *C. auris* were purified under native conditions using Ni-NTA affinity chromatography following the manufacturer’s instructions provided by Qiagen. For large scale bacterial cultures, primary inoculation of positive colonies were performed in 5 ml of LB media with appropriate antibiotics at 37°C and 220 rpm (Shukla and Rohatgi, 2020). Next day, secondary culture was inoculated in 500 ml of LB medium containing 100 μg/ml ampicillin and 25 μg/ml kanamycin at 37°C and 220 rpm. Protein expression was induced by adding 1 mM IPTG to mid-logarithmic phase cultures (OD_600_ ~0.6) for 8 hours at 18°C and 220 rpm. Bacteria were harvested by centrifugation at 10,000 rpm for 60 min and pellets were weighed and stored at -80°C. After freeze-thawing, the pellets were resuspended in lysis buffer (pH 8.0, 10 mM imidazole), containing lysozyme. Cell lysates were obtained through 10 cycles of sonication, each lasting 20 seconds with a 20-second interval between pulses. Subsequently, the lysates underwent centrifugation at 10,000 rpm for 30 min at 4°C and the recombinant proteins were purified from the supernatants using Ni-NTA affinity chromatography as per the manufacturer’s instructions provided by Qiagen. Briefly, the supernatants were gently mixed with Ni-NTA slurry for 1 hour at 4°C, and loaded onto Ni-NTA columns. After flowthrough collection, the columns were passed with Wash buffer I (pH 8.0, 20 mM imidazole) and Wash buffer II (pH 8.0, 50 mM imidazole). Finally, the protein samples were eluted using Elution buffer I (pH 8.0, 100 mM imidazole) and Elution buffer II (pH 8.0, 250 mM imidazole). The eluted samples containing purified recombinant Enolase proteins were collected and stored at 4°C. Aliquots of the flowthrough, wash and elution fractions were heat-denatured and analyzed on SDS PAGE and Coomassie staining as per established protocols. The elution fractions having pure proteins (free from impurities) were pooled together and subjected to dialysis for 12 hours at 4°C for removing imidazole. The dialysis buffer was changed at an interval of 4 hours. The dialysed and un-dialysed protein fractions were further analyzed on SDS-PAGE.

### Western blotting of recombinant enolase proteins from *C. albicans* and *C. auris*

The purified N-terminal histidine-tagged Enolase proteins were resolved on a 10% SDS-PAGE gel alongside a pre-stained protein marker (Bio Rad). His-tagged PspA protein was used as positive control along with BSA as negative control. After gel electrophoresis, they were transferred onto a nitrocellulose membrane (Bio-Rad). To prevent non-specific binding, the membrane was blocked with 3% (w/v) BSA in Tris-buffered saline (TBS) for 1 hour at room temperature. Subsequently, the membrane underwent four washes with TBS containing 0.05% Tween-20 (TBST). It was then incubated with an anti-His monoclonal antibody from Santa Cruz Biotechnology at a 1:1000 dilution for 1 hour at room temperature. After another four washes with TBST, the membrane was exposed to an HRP-conjugated goat anti-mouse IgG from Santa Cruz Biotechnology at a 1:5000 dilution for 1 hour at room temperature. After four washes with TBST and one wash with TBS, the membrane was exposed to a 3,3′-diaminobenzidine (DAB) substrate solution (0.5 mg/mL) at room temperature (Shukla et al., 2021a). The color development process was halted by rinsing with water. Subsequently, the blots were allowed to air-dry and scanned.

### CD spectroscopy of recombinant enolase proteins from *C. albicans* and *C. auris*

To assess the structural composition of the recombinant Enolase proteins, the dialyzed proteins from both *C. albicans* and *C. auris* were subjected to circular dichroism (CD) analysis using a Chirascan Circular Dichroism Spectrometer manufactured by Applied Photophysics Ltd., Surrey, UK (Shukla et al., 2021a). CD spectra were acquired using a 1 mm quartz cell with continuous nitrogen purging, spanning wavelengths from 190 to 250 nm in 1 nm increments, with an average acquisition time of 2.0 seconds at 18°C. The protein samples, prepared at a concentration of 1.5 mg/mL, were analyzed in a buffer consisting of 10 mM Tris at pH 7.4 with 100 mM NaCl. Every sample underwent three scans, which was then averaged. Following that, the baseline spectrum of the relevant buffer was subtracted to acquire the ultimate averaged spectrum. Afterward, plots specific to secondary structure were created by graphing the molar ellipticity in relation to the wavelength.

### Structural analysis of recombinant enolase proteins from *C. albicans* and *C. auris*

Tertiary 3D structure of Enolase protein from *C. auris* was modeled by using SWISS-MODEL server (https://swissmodel.expasy.org) based on available suitable homologous protein structure templates. The crystal structure of *C. albicans* Enolase protein (PDB ID: 7V67) was chosen as a template for modeling *C. auris* Enolase protein. The modeled 3D structure of *C. auris* Enolase protein and *C. albicans* Enolase protein were superimposed using PyMOL 3D structure visualization software (http://www.pymol.org/) for comparison of their respective secondary structure assessment (percentage of alpha helices, beta sheets and random coils) and surface accessibility (DeLano, 2002). The physical and chemical properties (including amino acid composition, molecular weight, theoretical isoelectric point, instability coefficient, aliphatic index and grand average of hydropathicity [GRAVY]) of both Enolase proteins were analyzed using ProtParam tool (http://web.expasy.org/protparam/). The alignment of the tertiary structures for the two proteins was conducted using PyMOL software, and the Root Mean Square Deviation (RMSD) values of the superimposed enolase proteins were assessed. The tertiary structures of Enolase proteins from both *C. albicans* and *C. auris* were validated through Ramachandran plots using the PROCHECK program available on the SAVES 6.0 structure validation server (https://saves.mbi.ucla.edu).

### B-cell epitope analysis of recombinant enolase proteins from *C. albicans* and *C. auris*

Linear B cell epitopes of Enolase proteins from *C. albicans* and *C. auris* were predicted by using *in silico* approach on BepiPred Linear Epitope Prediction 2.0 tool (http://tools.iedb.org/bcell/) available at IEDB web server (Jespersen et al., 2017). Amino acid sequences of both the Enolase proteins were selected, analyzed and the scoring threshold was set to 0.50.

For prediction of discontinuous B cell epitopes, a structure-based antibody prediction tool, DiscoTope (http://tools.iedb.org/discotope/), available at IEDB web server, was used to predict conformational B cell epitopes (Kringelum et al., 2012). This tool was used tertiary structure of Enolase protein from *C. albicans*, downloaded from RCSB PDB web server in PDB (PDB ID: 7V67) format (https://www.rcsb.org/structure/7V67). For conformational B cell epitopes prediction in *C. auris* Enolase protein, we used modeled tertiary structure from SWISS-MODEL in PDB format. For this analysis, a threshold of -3.7 was utilized to determine surface epitopes on both the Enolase proteins.

### T-cell epitope analysis of recombinant enolase proteins from *C. albicans* and *C. auris*

We used IEDB web server to predict T cell epitopes within Enolase proteins from *C. albicans* and *C. auris* for both the MHC class I and MHC class II alleles respectively.

For the prediction of MHC class I binding CD8 T-cell epitopes, we used IEDB-recommended 2023.09 NetMHCpan 4.1 EL method (http://tools.iedb.org/mhci/) (Jakhar and Gakhar, 2020). The MHC class I binding score >0.75 is regarded as excellent for T-cell epitopes (Jakhar and Gakhar, 2020; Shukla et al., 2022).

We also investigated the CD4 T-cell epitopes (MHC class II binding) in both Enolase proteins from *C. albicans* and *C. auris* respectively, using the IEDB-recommended 2023.09 NetMHCIIpan 4.1 EL tool available on (http://tools.iedb.org/mhcii/). It predicts the binding affinity of all possible epitopes with the available MHC II allele dataset. The predicted output is given in units of IC50 nM for combinatorial library and SMM align, where lower number indicates higher affinity. Predicted peptides with IC50 values <50 nM are considered high affinity, <500 nM intermediate affinity and <5000 nM low affinity (Shukla et al., 2022).

### Antigenicity and allergenicity prediction of enolase epitopes

The antigenicity values of linear B-cell and T-cell epitopes identified in Eno-albicans and Eno-auris proteins was predicted using VaxiJen server (version 2.0), (http://www.ddg-pharmfac.net/vaxijen/VaxiJen/VaxiJen.html). The antigenicity scores were obtained by selecting fungal target organism and using a default threshold value of 0.5. Epitopes exhibiting values higher than 0.5 were considered antigenic. The antigenic values of epitopes having less than 6 amino acid residues were not computed by VaxiJen (Doytchinova and Flower, 2007).

For predicting *in silico* allergenicity of the linear B-cell and T-cell epitopes identified in recombinant Enolase proteins from *C. albicans* and *C. auris*, AllerTOP server (version 2.0), (https://www.ddgpharmfac.net/AllerTOP/method.html) was used, selecting default parameters (Dimitrov et al., 2014).

## Results

### Cloning of recombinant enolase proteins from *C. albicans* and *C. auris*

**Figure 1:**
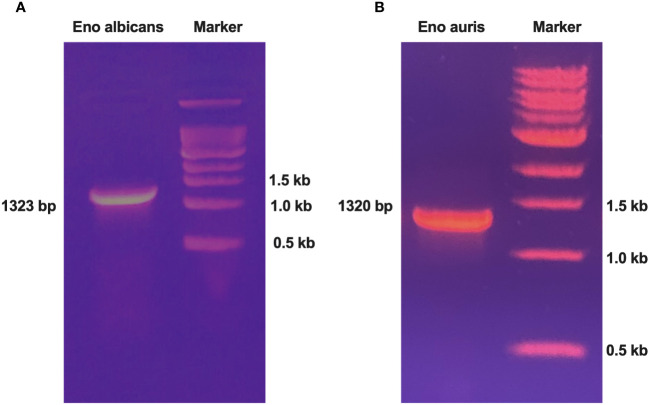
PCR amplification of Enolase genes from both *C albicans* and *C auris* strains. **(A)** PCR amplification of Enolase gene from *C albicans* (Eno-albicans: 1323 bp) and **(B)** PCR amplification of Enolase gene from *C auris* (Eno-auris: 1320 bp). Marker: 1 kb DNA ladder (last three bands of ladder are depicted).

Genomic DNA of the *Candida* species, *C. albicans* strain SC5314 (ATCC MYA-2876), and *C. auris* South Asian strain (Clade 1 CBS 470280) were extracted using a commercial kit as per manufacturer’s instructions. Gene specific primers were designed for amplifying the full-length Enolase genes. The forward and reverse primers were incorporated with unique restriction enzymes (BamH1 and PstI, respectively) for ensuring directional cloning, as outlined in **Table 1**. PCR amplification of full-length Enolase genes was performed using genomic DNA of *C. albicans* and *C. auris.* The amplicon sizes obtained for *C. albicans*, and *C. auris* Enolase genes were 1323 bp and 1320 bp, respectively (**Figure 1A, B**). After PCR amplification, the resulting amplicons were digested using BamH1 and PstI restriction enzymes and cloned into double-digested pQE30Xa vector, ensuring compatibility for directional cloning. The ligated constructs were then transformed into competent cells of *E. coli* XL-1 Blue strain. Colonies obtained post-transformation were cultured for plasmid isolation, and positive clones were identified through restriction analysis and verified by sequencing.

### Sequence alignment of recombinant enolase proteins from *C. albicans* and *C. auris*

**Figure 2:**
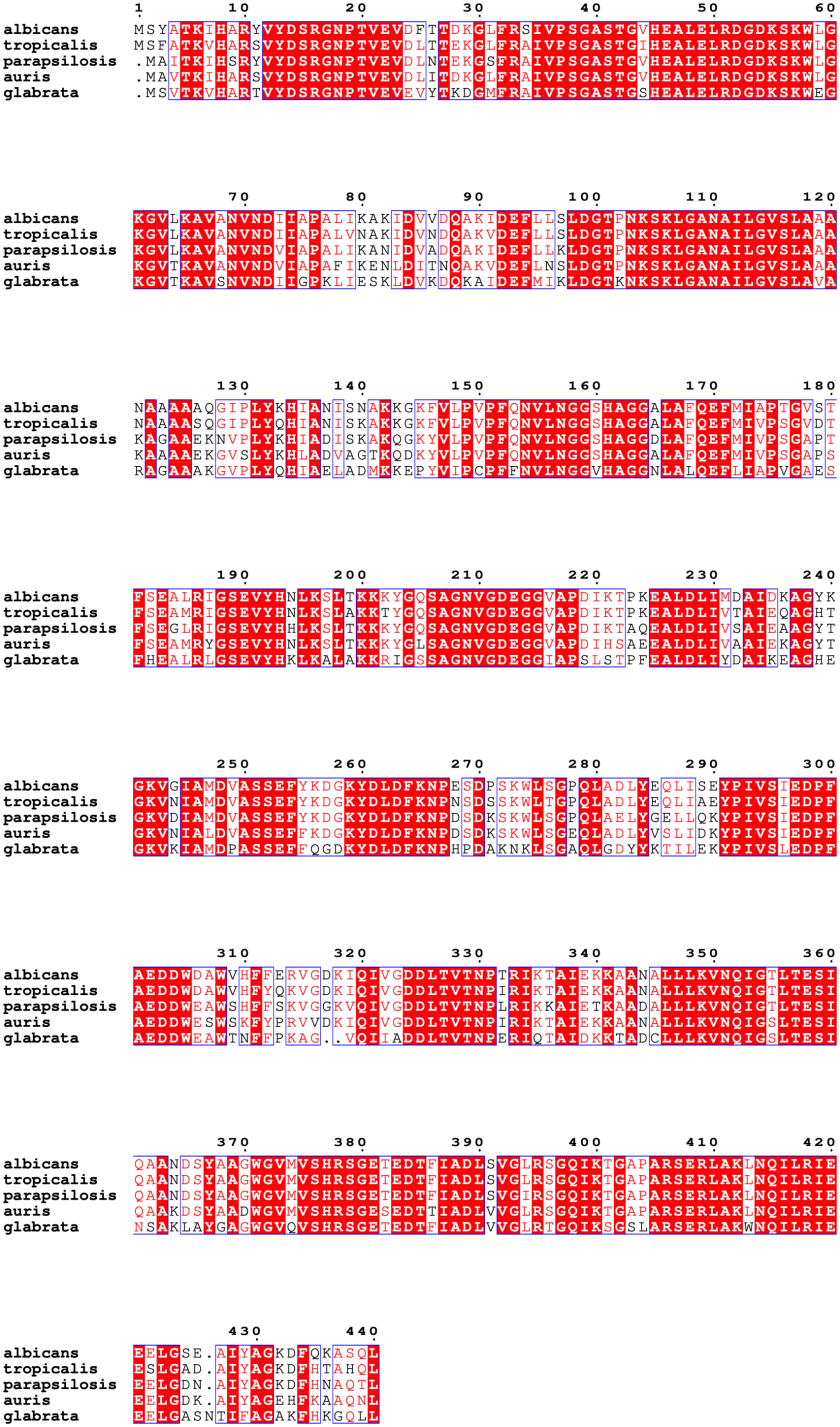
Multiple sequence alignment of full-length Enolase proteins from related *Candida* species. Amino acid sequences of Enolase from *C. albicans* (accession number: AAB46358.1) and *C. auris* (accession number: KNE00479.1) were aligned by Clustal Omega and are depicted using ESPript 3.0. Amino acid positions of Eno-albicans (1–440) and Eno-auris (1–439) are indicated and numbered according to Enolase of *C. albicans*. Universally conserved amino acid residues are depicted in white and marked with red highlights. Residues exhibiting a conservation rate exceeding 70% are enclosed within a blue box and indicated in red. Altered non-identical residues are unboxed and written in black.

The sequence alignment of Enolase proteins from *C. albicans* (Genbank accession number: AAB46358.1), *C. auris* (Genbank accession number KNE00479.1), *C. tropicalis* (Genbank accession number EER32738.1), *C. parapsilosis* (Genbank accession number CCE43078.1) and *C. glabrata* (Genbank accession number CAG59252.1) was performed using Clustal Omega and ESPRIPT 3. A high degree of homology was seen in Enolase sequences among these five *Candida* species. CLUSTAL alignment showed >80% identity between Enolase proteins between *C. albicans* and *C. auris* (**Figure 2**). Out of a total of 440 amino acids, 359 amino acids were identical between Eno-albicans and Eno-auris (81.6%), 29 amino acids were near identical (6.6%), and 52 amino acids were non-identical (11.8%).

### Expression and purification of recombinant enolase proteins from *C. albicans* and *C. auris*

**Figure 3:**
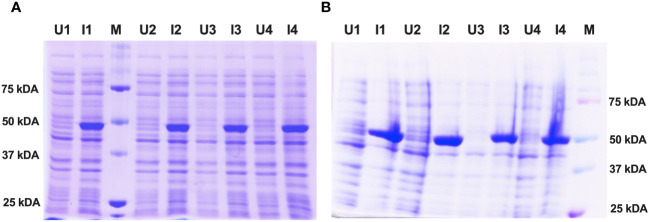
Expression and induction check of recombinant Enolase proteins from *C albicans* and *C auris*. **(A)** Induction of Eno-albicans protein: ~51 kDa; 440 aa. **(B)** Induction of Eno-auris protein: ~51 kDa; 439 aa. Lanes U: Uninduced samples from 4 bacterial colonies; Lanes I: Induced samples from 4 bacterial colonies; M: Protein Marker, Marker bands indicated on right.

For protein expression, the histidine-tagged fusion protein constructs of Enolase belonging to *C. albicans* and *C. auris* were re-transformed in *E. coli* strain SG13009 (Qiagen). Small-scale *E. coli* bacterial cultures were then subjected to IPTG induction with continuous agitation using an orbital shaker. The optimal protein expression conditions were achieved at an IPTG concentration of 1 mM, a cell density (OD_600_) of 0.6 and an induction duration of 8 hours at 18°C. Following induction, both the un-induced and induced bacterial cultures were analyzed using SDS-PAGE electrophoresis. The induced Enolase proteins exhibited specific bands at approximately 51 kDa, consistent with the expected molecular weight of Enolase fusion proteins from both *C. albicans* and *C. auris* (**Figure 3A, B**).

**Figure 4:**
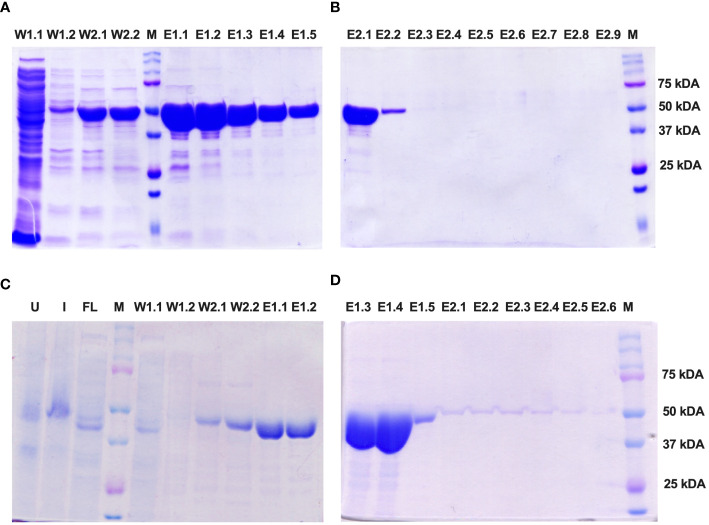
SDS-PAGE analysis of purified His-tagged Enolase proteins used in the study. Protein fractions obtained under native conditions (50 mM NaH_2_PO_4_, 300 mM NaCl, pH 8.0) with different concentrations of imidazole: 10 mM (Lysis), 20 mM (Wash 1), 50 mM (Wash 2), 100 mM (Elution 1) and 250 mM (Elution 2). **(A, B)** Fractions of Eno-albicans protein (440 aa; ~51 kDa). **(C, D)** Fractions of Eno-auris protein (439 aa; ~51 kDa). Lane U: Uninduced sample; Lane I: Induced sample; Lane FL: Flow through; Lanes W1-W2: Wash fractions; Lanes E1.1 to E1.5: five sequentially eluted fractions with E1 buffer; Lanes E2.1 to E2.5: five sequentially eluted fractions with E2 buffer; M: Protein Marker, Marker bands indicated on right.

The Enolase proteins from both *C. albicans* and *C. auris* were subsequently purified using Ni-NTA affinity chromatography following the manufacturer’s instructions (Qiagen) under soluble, native conditions. SDS PAGE analysis revealed fractions of the purified Eno-albicans protein obtained after passing the Ni-NTA column with wash (W1 and W2) and elution (E1 and E2) buffers (**Figure 4A, B**). The eluates of the purified Eno-auris protein recovered after passing the Ni-NTA column with wash (W1 and W2) and elution (E1 and E2) buffers was also analyzed on SDS PAGE (**Figure 4C, D**). The purified his-tagged Eno-albicans and Eno-auris proteins were obtained at approximately 51 kDa.

### Western blotting of recombinant enolase proteins from *C. albicans* and *C. auris*

**Figure 5:**
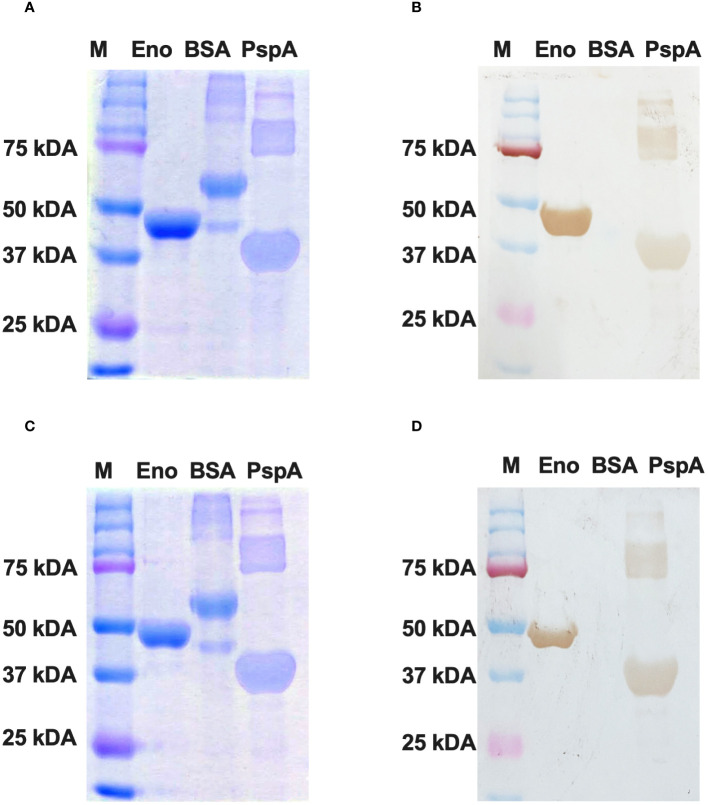
Western blotting of recombinant Enolase proteins used in the study. **(A)** SDS PAGE using Coomassie Brilliant Blue staining of Enolase protein from *C albicans*. **(B)** Western Blot of Eno-albicans protein using DAB substrate. **(C)** SDS PAGE using CBR staining of Enolase protein from *C auris*. **(B)** Western Blot of Eno-auris protein using DAB substrate. Histidine-tagged Eno-albicans and Eno-auris proteins obtained at ~51 kDa. PspA and BSA proteins are used as positive and negative controls, respectively. Protein Marker bands are indicated on right.

The purified Enolase protein samples belonging to *C. albicans* and *C. auris* were stored at 4°C and subjected to dialysis for 12 hours at 4°C with an interval of 4 hours for changing the dialysis buffer. After dialysis, the purified Enolase fusion proteins (~51 kDa) were analyzed on SDS PAGE by Coomassie Blue staining (**Figure 5A, C**), transferred on nitrocellulose membranes, and confirmed through Western blot analysis using the anti-His antibody along with appropriate negative (BSA) and positive (PspA) controls (**Figure 5B, D**).

### CD spectroscopy of recombinant enolase proteins from *C. albicans* and *C. auris*

**Figure 6:**
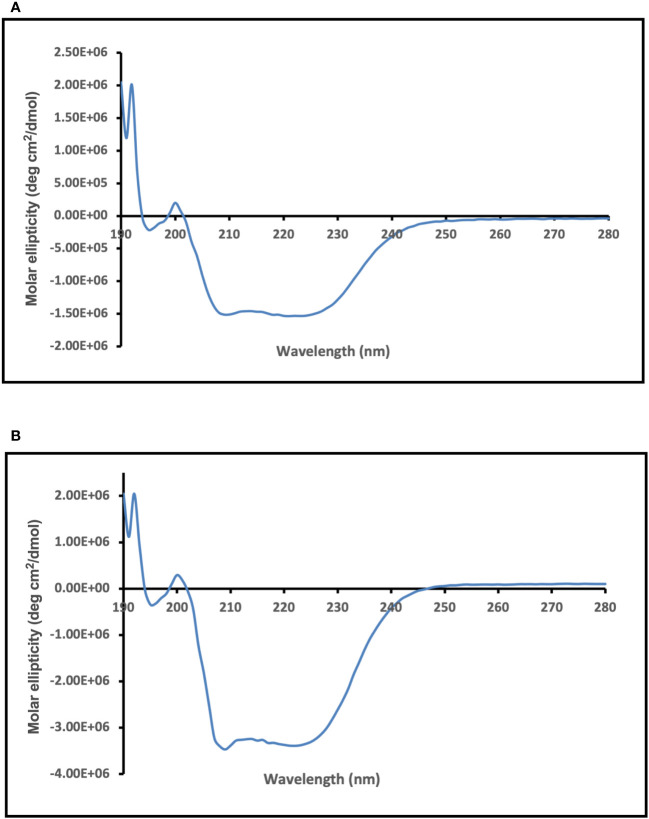
CD spectra analysis of purified and dialyzed recombinant Enolase protein samples used in the study. CD spectra obtained for **(A)** Eno-albicans and **(B)** Eno-auris proteins is shown. Molar ellipticity is depicted on the y-axis with wavelength on x-axis.

CD spectroscopy was used to assess the secondary structure of the dialyzed Enolase proteins. The CD spectral data underwent analysis and deconvolution utilizing an online CD spectra algorithm. The CD spectra obtained for dialysed forms of the Enolase proteins from *C. albicans* (**Figure 6A**) and *C. auris* are shown (**Figure 6B**). Molar ellipticity is illustrated on the y-axis, while the wavelength is depicted on the x-axis. The CD spectra of yeast Enolase is characterized by double minimum (209 nm, 222 nm) and maximum (192 nm) peaks. The CD spectra analysis (**Figure 6**) of the dialyzed Enolase protein samples show presence of dip at around 209 nm and 220 nm, indicating existence of secondary structure elements, which are primarily α-helical. Overall, the CD spectra curves of Enolase proteins exhibits signature of an α-helix protein.

### Structural analysis of recombinant enolase proteins from *C. albicans* and *C. auris*

**Figure 7:**
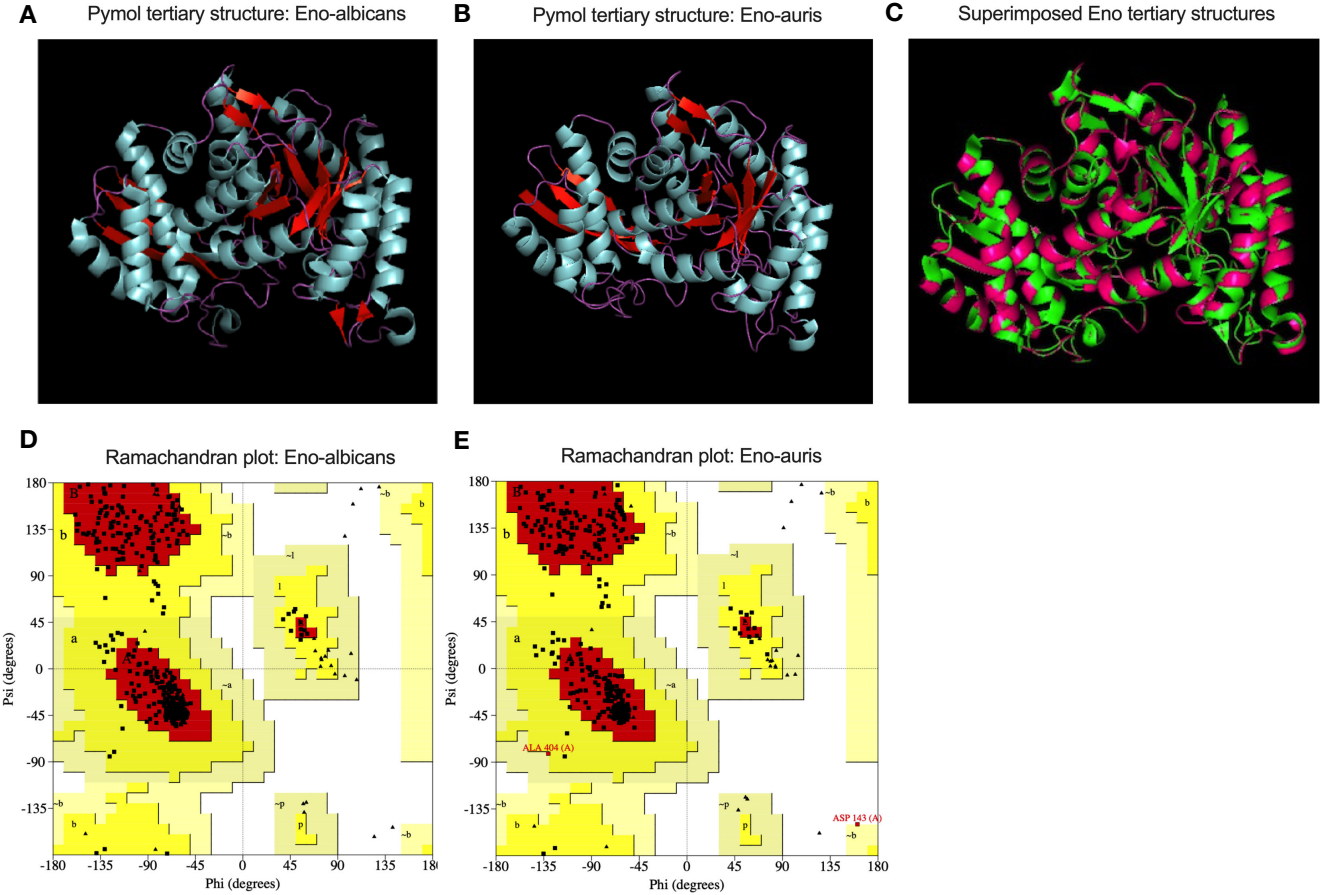
A cartoon view of the tertiary structural models of **(A)** Eno-albicans and **(B)** Eno-auris proteins is shown using Pymol. The amino acid residues corresponding to alpha helix, beta sheets and random coils are shown in cyan, red, and magenta, respectively, for both the Enolase proteins. **(C)** Superimposed tertiary structures of Eno-albicans and Eno-auris. **(D)** Ramachandran plot for Eno-albicans **(E)** Ramachandran plot for Eno-auris.

The primary structures of both the recombinant Enolase proteins from *C. albicans* and *C. auris* were analyzed, and different parameters were computed using ExPasy ProtParam tool (**Table 2**). Amino acid sequence analysis provided valuable insights of amino acid composition, stability, hydrophobicity, and immunogenic properties of Enolase proteins from *C. albicans* and *C. auris* respectively. Upon comparing the physicochemical properties of both Enolase proteins, we observed minor differences in their isoelectric point (pI), instability index, aliphatic index and GRAVY score. The theoretical isoelectric point (pI) was found to be lower for *C. auris* Enolase protein (5.44) when compared to *C. albicans* Enolase protein (5.54). Additionally, *C. auris* Enolase protein (29.12) was classified as more stable than *C. albicans* Enolase protein (32.91) due to its low instability index. Aliphatic index is associated with increase in the thermostability of proteins, a higher aliphatic index value indicating more stability. As per their aliphatic index values, Eno-auris (93.83) protein was found to be more thermostable than the Eno-albicans (92.09) protein. Furthermore, we used GRAVY score to compare the hydrophobicity of both the Enolase proteins. We found negative GRAVY values for both Enolase proteins from *C. albicans* and *C. auris*, indicating their hydrophilic nature. Nevertheless, Eno-auris (-0.195) protein exhibits lower hydrophobicity score than the Eno-albicans (-0.192) protein, suggesting its higher probability of being exposed on the protein surface, and may be more accessible to antibodies and immune cells. The 3D structure of Enolase protein from *C. auris* was generated using SWISS-MODEL server. The tertiary structure of both Eno-albicans and Eno-auris proteins were visualized using PyMol software (**Figure 7A, B**). It was observed that Enolase protein from *C. albicans* is predominantly alpha helical, exhibiting the following composition: alpha helix (50.45%), beta sheets (16.36%) and random coils (33.18%). The secondary structure of Enolase protein from *C. auris* was similar to that of Eno-albicans, having the following composition: alpha helix (43.50%), beta sheets (21.64%), and random coils (34.85%) (**Table 2**). Both Eno-albicans and Eno-auris proteins had predominantly alpha helices, (indicated in cyan coloration) in their structures. Beta sheets (depicted in red), also contributes to the protein’s overall structural stability. The random coils regions, (depicted in magenta), connected the alpha helices and beta sheets. The tertiary structures of the two Enolase proteins were superimposed to assess homology (**Figure 7C**). The superimposed tertiary structures of the two Enolase proteins were aligned using PyMOL, revealing a RMSD value of 0.054 Å. This value indicates a relatively close structural similarity between the proteins (Kosloff and Kolodny, 2008). The tertiary structures of the two proteins were validated using PROCHECK analysis. The PROCHECK analysis produced Ramachandran plots for both Eno-albicans and Eno-auris proteins (**Figure 7D, E**). The plot statistics revealed that 91.3% and 91.4% of the residues were localized in the most favored regions [A, B, L] for Eno-albicans and Eno-auris, respectively. Additionally, 8.7% and 8.1% of residues were situated in the additional allowed regions [a, b, l, p] for Eno-albicans and Eno-auris, respectively. Ideally, a good quality model is expected to have over 90% of its residues in the most favored regions (Laskowski et al., 1996).

**Table 2:** Analysis of physicochemical properties of *C. albicans* and *C. auris* Enolase proteins using ProtParam server.

Criteria	*C. albicans* Enolase	*C. auris* Enolase
Number of amino acids	440	439
Molecular weight (Daltons)	47231.53	47107.28
Theoretical pI	5.54	5.44
Instability index	32.91 (stable protein)	29.12 (stable protein)
Aliphatic index	92.09	93.83
Grand average of hydropathicity (GRAVY)	-0.192 (hydrophilic protein)	-0.195 (hydrophilic protein)
Secondary Structure	Amino acids in Eno-albicans	Amino acids in Eno-auris
Alpha Helix	222 (50.45%)	191 (43.50%)
Beta Sheets	72 (16.36%)	95 (21.64%)
Random coils	146 (33.18%)	153 (34.85%)

### B-cell epitope analysis of recombinant enolase proteins from *C. albicans* and *C. auris*

The amino acid sequences of both *C. albicans* and *C. auris* Enolase proteins were computed for prediction of linear sequential B cell epitopes on BepiPred 2.0 server, using a threshold value of 0.5. The same threshold value was applied for the prediction of linear B-cell epitopes on the BepiPred-2.0 server for both the *C. albicans* Enolase protein and *C. auris* Enolase protein, resulting in the identification of 16 epitopes for each protein (**Table 3**). The analysis provided length and position of each linear B cell epitopes, which are outlined in **Table 3**. Antigenicity analysis of linear B-cell epitopes conducted via the VaxiJen server identified 8 epitopes in the Enolase protein from *C. albicans* and 7 epitopes in the Enolase protein from *C. auris*. Additionally, the assessment of linear B-cell epitope allergenicity using the AllerTOP server predicted 3 allergenic epitopes in the Enolase protein from *C. albicans* and 4 allergenic epitopes in the Enolase protein from *C. auris*.

**Table 3:** Linear B-cell epitopes of Enolase proteins predicted using Bepipred 2.0 server.

S.N.	Enolase (*C. albicans*)	Length	Position	Antigenicity*	Allergenicity*	Enolase (*C. auris*)	Length	Position	Antigenicity*	Allergenicity*
1	DSRG	4	14–17	–	No	DSRG	4	13–16	–	No
2	GASTGVH	7	39–45	3.9366	No	ASTGVHEA	8	39–46	-0.4341	No
3	DGDKSKWLGK	10	52–61	3.1536	No	LRDGDKSKWLGKG	13	49–61	0.1838	No
4	IDVVDQAK	8	83–90	-0.3021	Allergen	LDITNQAK	8	82–89	0.2072	Allergen
5	LDGTPNKSKL	10	98–107	-0.3536	No	SLDGTPNKSKL	11	96–106	0.1867	No
6	NISNAKKGK	9	137–145	1.5783	Allergen	DVAGTKQDKY	10	136–145	1.3056	No
7	GGSHAGG	7	158–164	2.2261	No	GSHAGG	6	158–163	3.7643	No
8	KKKYGQSAGNV	11	200–210	1.9044	No	KKKYGLSAGN	10	199–208	2.0057	No
9	KTPKE	5	221–225	–	No	IHSAEE	6	219–224	1.0220	Allergen
10	AGYK	4	237–240	–	No	YT	2	238–239	–	No
11	YKDGKYDLDFKNPESDPSKWLSGPQLAD	28	255–282	1.0260	No	KDGKYDLDFKNPDSDKSKWLSGEQLADL	28	255–282	1.0672	Allergen
12	AEDDWDA	7	301–307	0.3402	No	AEDDWESWSK	10	300–309	0.9545	No
13	V	1	309–309	–	No	A	1	336–336	–	No
14	PTRIKTA	7	331–337	1.3135	No	GESED	5	379–383	–	Allergen
15	GETE	4	380–383	–	Allergen	D	1	424–424	–	No
16	AGKDFQKA	8	430–437	1.5140	No	IYAGEHFKA	9	427–435	1.1662	No

*Antigenicity and Allergenicity values predicted by VaxiJen and AllerTOP servers, respectively.

Epitopes having less than 6 amino acids not computed by VaxiJen, and values depicted by ‘-’.

Default threshold of 0.5 used for computing antigenicity, values higher than threshold are considered antigenic.

Conformation-based B-cell epitopes were computed on DiscoTope 2.0 server available at IEDB web server, using tertiary structure of both the Enolase proteins. A threshold value of − 3.7 was chosen for the computation, which corresponds to default value. The analysis provided residue ID, residue name, contact number, propensity score, and DiscoTope score for each amino acid meeting the threshold value (**Table 4**). This tool predicted 23 and 16 discontinuous B cell epitopes in *C. albicans* Enolase and *C. auris* Enolase proteins, respectively (**Table 4**).

**Table 4:** Discontinuous B-cell epitopes of Enolase proteins predicted using DiscoTope 2.0 server.

S.N.	*C. albicans* Enolase					*C. auris* Enolase				
	Residue ID	Residue Name	Contact Number	Propensity Score	DiscoTope Score	Residue ID	Residue Name	Contact Number	Propensity Score	DiscoTope Score
1	17	GLY	3	-1.208	-1.414	16	GLY	6	-1.075	-1.642
2	53	GLY	6	-2.759	-3.131	52	GLY	7	-1.968	-2.547
3	54	ASP	10	-2.062	-2.975	53	ASP	11	-1.07	-2.212
4	55	LYS	0	-0.287	-0.254	54	LYS	0	0.529	0.469
5	56	SER	0	1.095	0.969	55	SER	0	1.919	1.698
6	57	LYS	12	-1.504	-2.711	56	LYS	12	-0.68	-1.982
7	58	TRP	19	-1.502	-3.514	57	TRP	20	-1.098	-3.272
8	59	LEU	4	-1.475	-1.765	58	LEU	5	-0.025	-0.597
9	60	GLY	2	-1.611	-1.656	59	GLY	2	-0.645	-0.801
10	61	LYS	17	-1.887	-3.625	60	LYS	17	-1.68	-3.442
11	163	GLY	6	-2.861	-3.222	256	ASP	10	-1.743	-2.692
12	204	GLY	7	-2.345	-2.88	266	PRO	0	-1.053	-0.932
13	205	GLN	0	-2.282	-2.02	267	ASP	0	0.213	0.188
14	206	SER	5	-2.148	-2.476	269	ASP	3	-1.3	-1.495
15	257	ASP	10	-1.213	-2.223	270	LYS	6	-0.732	-1.338
16	258	GLY	1	-3.052	-2.816	271	SER	0	-1.861	-1.647
17	267	PRO	1	-0.376	-0.447	–	–	–	–	–
18	268	GLU	0	1.105	0.977	–	–	–	–	–
19	269	SER	22	0.682	-1.926	–	–	–	–	–
20	270	ASP	2	0.785	0.465	–	–	–	–	–
21	271	PRO	3	0.857	0.413	–	–	–	–	–
22	272	SER	0	-0.032	-0.028	–	–	–	–	–
23	273	LYS	14	-1.112	-2.594	–	–	–	–	–

### T-cell epitope analysis of recombinant enolase proteins from *C. albicans* and *C. auris*

Because MHC binding is essential for T-cell-mediated immune responses to antigenic peptides, we employed it as a parameter for forecasting potential antigenic peptides within recombinant Enolase proteins from *C. albicans* and *C. auris*, in our study.

To identify the CD8 T-cell epitopes binding to human MHC class I alleles, we employed the NetMHCpan EL 4.1 method (http://tools.iedb.org/mhci/, version 2023.09), as recommended by IEDB to forecast the MHC class I binding affinity of T-cell epitopes for the human MHC I alleles accessible on the IEDB server. This tool predicts the binding affinity of each peptide with various MHC alleles. We found 23,301 and 11,546 CD8 T-cell potential peptides in Enolase proteins from *C. albicans* and *C. auris*, respectively for 22 MHC I alleles. According to published data, binding score >0.75 for the MHC class I in T-cell epitopes is regarded as strong binders (Jakhar and Gakhar, 2020; Shukla et al., 2022). We identified 38 and 42 potential MHC I binding CD8 T-cell epitopes in Enolase proteins from *C. albicans* and *C. auris*, respectively, whose score value was >0.75 (**Table 5**). The Enolase protein from *C. auris* exhibits a higher number of CD8 T-cell epitopes for MHC I alleles compared to *C. albicans* Enolase protein. This finding indicates that the Enolase protein from *C. auris* might possess more antigenic properties or a higher immunogenicity potential for MHC I presentation than the Enolase protein from *C. albicans*. Evaluation of CD8 T-cell epitope antigenicity through the VaxiJen server revealed 16 epitopes in the Enolase protein from *C. albicans* and 19 epitopes in the Enolase protein from *C. auris*. Meanwhile, CD8 T-cell epitope allergenicity analysis conducted via the AllerTOP server predicted 21 allergenic epitopes in the Enolase protein from *C. albicans* and 23 allergenic epitopes in the Enolase protein from *C. auris*.

**Table 5:** CD8 T-cell epitopes of Enolase proteins predicted using IEDB server.

S.N	Peptide (Eno-Albicans)	Position (aa)	Human MHC I alleles	Antigenicity*	Allergenicity*	S.N	Peptide (Eno-Auris)	Position (aa)	Human MHC I alleles	Antigenicity*	Allergenicity*
1	MSYATKIHAR	1–10	HLA-A*68:01	1.8389	No	1	MAVTKIHAR	1–9	HLA-A*68:01, HLA-A*33:01	0.3297	Allergen
2	SYATKIHAR	2–10	HLA-A*33:01, HLA-A*31:01	2.4177	No	2	TGVHEALELR	41–50	HLA-A*68:01	1.2790	No
3	YATKIHARY	3–11	HLA-B*35:01	1.9411	No	3	DVIAPAFIK	71–79	HLA-A*68:01	-0.6924	No
4	FTTDKGLFR	25–33	HLA-A*68:01	0.0858	No	4	APAFIKENL	74–82	HLA-B*07:02	-0.8746	No
5	TGVHEALELR	42–51	HLA-A*68:01	1.2790	No	5	KVDEFLNSL	89–97	HLA-A*02:06, HLA-A*02:01	-0.6870	No
6	DIIAPALIK	72–80	HLA-A*68:01	-1.2823	Allergen	6	AAEKGVSLY	123–131	HLA-A*01:01	0.9198	No
7	IIAPALIKA	73–81	HLA-A*02:06	-1.6139	No	7	SLYKHLADV	129–137	HLA-A*02:03, HLA-A*02:01	0.8184	No
8	APALIKAKI	75–83	HLA-B*07:02	-1.3270	Allergen	8	KHLADVAGTK	132–141	HLA-A*03:01	2.1790	No
9	KIDEFLLSL	90–98	HLA-A*02:06, HLA-A*02:01	0.7107	Allergen	9	YVLPVPFQNV	145–154	HLA-A*02:06	0.3473	Allergen
10	LSLDGTPNK	96–104	HLA-A*11:01	0.6941	No	10	LPVPFQNVL	147–155	HLA-B*35:01, HLA-B*51:01, HLA-B*07:02, HLA-B*53:01	-0.3587	Allergen
11	AAAQGIPLY	124–132	HLA-B*35:01, HLA-A*30:02	0.9126	Allergen	11	AMRYGSEVY	183–191	HLA-B*15:01	-0.2073	No
12	AAAQGIPLYK	124–133	HLA-A*11:01, HLA-A*03:01	0.3762	Allergen	12	RYGSEVYHNL	185–194	HLA-A*24:02	-1.3007	No
13	AAQGIPLYK	125–133	HLA-A*11:01, HLA-A*03:01	0.0940	Allergen	13	SEVYHNLKSL	188–197	HLA-B*40:01	-0.6079	Allergen
14	HIANISNAK	134–142	HLA-A*68:01	1.6629	No	14	EVYHNLKSL	189–197	HLA-A*68:02	0.0334	Allergen
15	FVLPVPFQNV	146–155	HLA-A*02:06	0.4998	Allergen	15	GVAPDIHSA	214–222	HLA-A*02:06	2.0207	
16	LPVPFQNVL	148–156	HLA-B*35:01, HLA-B*51:01, HLA-B*07:02, HLA-B*53:01	-0.3587	Allergen	16	DVASSEFFK	247–255	HLA-A*68:01	0.5734	Allergen
17	MIAPTGVSTF	172–181	HLA-B*15:01	-0.6545	No	17	SSEFFKDGKY	250–259	HLA-A*01:01	-2.5953	Allergen
18	ALRIGSEVY	184–192	HLA-B*15:01	0.1902	Allergen	18	SEFFKDGKY	251–259	HLA-B*44:03	-2.9801	Allergen
19	SEVYHNLKSL	189–198	HLA-B*40:01	-0.6079	Allergen	19	NPDSDKSKW	265–273	HLA-B*53:01	1.7531	
20	EVYHNLKSL	190–198	HLA-A*68:02	0.0334	Allergen	20	LSGEQLADLY	274–283	HLA-A*01:01	0.7552	Allergen
21	GVAPDIKTPK	215–224	HLA-A*11:01	2.4183		21	QLADLYVSL	278–286	HLA-A*02:01, HLA-A*02:03, HLA-A*02:06	0.6934	Allergen
22	DAIDKAGYK	232–240	HLA-A*68:01	-1.8672	Allergen	22	SLIDKYPIV	285–293	HLA-A*02:03, HLA-A*02:01, HLA-A*02:06	1.3721	No
23	AMDVASSEFY	246–255	HLA-A*01:01	0.8279	Allergen	23	FAEDDWESW	299–307	HLA-B*53:01, HLA-B*58:01	1.2471	No
24	DVASSEFYK	248–256	HLA-A*68:01	0.3548	Allergen	24	ESWSKFYPR	305–313	HLA-A*33:01, HLA-A*68:01	0.6127	Allergen
25	SSEFYKDGKY	251–260	HLA-A*01:01	-2.2849	Allergen	25	KFYPRVVDK	309–317	HLA-A*30:01	-1.8750	Allergen
26	SEFYKDGKY	252–260	HLA-B*44:03	-2.6647	Allergen	26	FYPRVVDKI	310–318	HLA-A*24:02	-2.7299	Allergen
27	NPESDPSKW	266–274	HLA-B*53:01	1.8939	No	27	RVVDKIQIV	313–321	HLA-A*02:06, HLA-A*02:03	-0.3037	No
28	QLADLYEQL	279–287	HLA-A*02:01, HLA-A*02:06, HLA-A*02:03	0.1721	No	28	TVTNPIRIK	326–334	HLA-A*68:01	1.5574	Allergen
29	DLYEQLISEY	282–291	HLA-A*26:01	1.0256	No	29	NPIRIKTAI	329–337	HLA-B*07:02	0.4566	Allergen
30	DAWVHFFER	306–314	HLA-A*33:01, HLA-A*68:01	1.5022	Allergen	30	RIKTAIEKK	332–340	HLA-A*30:01, HLA-A*03:01	-0.0444	Allergen
31	RIKTAIEKK	333–341	HLA-A*30:01, HLA-A*03:01	-0.0444	Allergen	31	IEKKAANAL	337–345	HLA-B*40:01	-0.0995	No
32	IEKKAANAL	338–346	HLA-B*40:01	-0.0995	No	32	KAANALLLK	340–348	HLA-A*11:01, HLA-A*03:01	0.6859	No
33	KAANALLLK	341–349	HLA-A*11:01, HLA-A*03:01	0.6859	No	33	SLTESIQAA	354–362	HLA-A*02:03	1.7409	Allergen
34	FIADLSVGL	386–394	HLA-A*02:06, HLA-A*02:01, HLA-A*02:03, HLA-A*68:02	2.1232	Allergen	34	SEDTTIADL	381–389	HLA-B*40:01	1.0000	Allergen
35	FIADLSVGLR	386–395	HLA-A*68:01	1.6722	Allergen	35	TTIADLVVGL	384–393	HLA-A*68:02	0.7196	No
36	KTGAPARSER	400–409	HLA-A*31:01	-0.7044	Allergen	36	TIADLVVGL	385–393	HLA-A*68:02, HLA-A*02:06, HLA-A*02:01	0.3288	No
37	EELGSEAIY	421–429	HLA-B*44:03	0.0277	No	37	TIADLVVGLR	385–394	HLA-A*68:01	0.4506	No
38	AIYAGKDFQK	427–436	HLA-A*03:01, HLA-A*11:01	1.1685	No	38	KTGAPARSER	399–408	HLA-A*31:01	-0.7044	Allergen
						39	EELGDKAIY	420–428	HLA-B*44:03, HLA-B*44:02	-1.2705	Allergen
						40	KAIYAGEHFK	425–434	HLA-A*03:01	0.5602	Allergen
						41	AIYAGEHFK	426–434	HLA-A*11:01, HLA-A*03:01	0.4248	Allergen
						42	GEHFKAAQNL	430–439	HLA-B*40:01	2.2407	Allergen

*Antigenicity and Allergenicity values predicted by VaxiJen and AllerTOP servers, respectively.

For identifying CD4 T-cell epitopes binding to MHC II alleles, we performed *in silico* analysis of both *C. albicans* and *C. auris* Enolase proteins using IEDB-recommended NetMHCIIpan 4.1 EL tool available on (http://tools.iedb.org/mhcii/, version 2023.09). The server predicted a total of 1477 and 1436 T-cell peptides in *C. albicans* and *C. auris* Enolase proteins, respectively for 13 MHC II binding alleles. By establishing a threshold below 50 nM IC50, we detected a combined total of 129 and 121 possible MHC II binding CD4 T-cell epitopes in *C. albicans* and *C. auris* Enolase proteins respectively, with good binding affinities (**Table 6**). The server prediction indicates that the CD4 T-cell epitopes for MHC II alleles were found to be comparable in both the Enolase proteins from *C. albicans* and *C. auris*. Such comparability in MHC II epitopes may imply similarities in immune responses or interactions with host immune cells mediated by MHC II presentation, between *C. albicans* and *C. auris* infections. Evaluation of CD4 T-cell epitope antigenicity through the VaxiJen server revealed 77 epitopes in the Enolase protein from *C. albicans* and 69 epitopes in the Enolase protein from *C. auris*. Meanwhile, CD8 T-cell epitope allergenicity analysis conducted via the AllerTOP server predicted 58 allergenic epitopes in the Enolase protein from *C. albicans* and 37 allergenic epitopes in the Enolase protein from *C. auris*.

**Table 6:** CD4 T-cell epitopes of Enolase proteins predicted using IEDB server.

SN	Human MHC II alleles	Peptide (Eno-albicans)	Position (aa)	Antigenicity*	Allergenicity*	SN	Human MHC II alleles	Peptide (Eno-auris)	Position (aa)	Antigenicity*	Allergenicity*
1	HLA-DQA1*01:02/ DQB1*06:02	VSLAAANAAAAAQGI	115–129	0.7498	No	1	HLA-DQA1*01:02/ DQB1*06:02	GVSLAAAKAAAAEKG	113–127	0.9995	Allergen
		GVSLAAANAAAAAQG	114–128	1.1569	Allergen			AILGVSLAAAKAAAA	110–124	1.1800	No
		ILGVSLAAANAAAAA	112–126	1.1319	No			ILGVSLAAAKAAAAE	111–125	1.4324	Allergen
		LGVSLAAANAAAAAQ	113–127	1.4840	No			LGVSLAAAKAAAAEK	112–126	1.7272	Allergen
		AILGVSLAAANAAAA	111–125	0.9584	No			VSLAAAKAAAAEKGV	114–128	0.6287	Allergen
		SLAAANAAAAAQGIP	116–130	0.5159	Allergen						
		LAAANAAAAAQGIPL	117–131	0.5159	Allergen						
2	HLA-DQA1*05:01/ DQB1*03:01	ILGVSLAAANAAAAA	112–126	1.1319	No	2	HLA-DQA1*05:01/ DQB1*03:01	ILGVSLAAAKAAAAE	111–125	1.4324	No
		LGVSLAAANAAAAAQ	113–127	1.4840	No			LGVSLAAAKAAAAEK	112–126	1.7272	No
		GVSLAAANAAAAAQG	114–128	1.1569	Allergen			GVSLAAAKAAAAEKG	113–127	0.9995	Allergen
		VSLAAANAAAAAQGI	115–129	0.7498	No			VSLAAAKAAAAEKGV	114–128	0.6287	Allergen
		AILGVSLAAANAAAA	111–125	0.9584	No			AILGVSLAAAKAAAA	110–124	1.1800	No
		SLAAANAAAAAQGIP	116–130	0.5159	Allergen			SLAAAKAAAAEKGVS	115–129	0.9137	Allergen
		LAAANAAAAAQGIPL	117–131	1.1122	Allergen			LAAAKAAAAEKGVSL	116–130	1.1875	No
		NAILGVSLAAANAAA	110–124	1.1003	No			NAILGVSLAAAKAAA	109–123	1.2659	No
		AAANAAAAAQGIPLY	118–132	0.8205	Allergen			NVLNGGSHAGGALAF	153–167	0.7670	Allergen
		ANAILGVSLAAANAA	109–123	1.1338	No			VLNGGSHAGGALAFQ	154–168	0.3967	Allergen
		NVLNGGSHAGGALAF	154–168	0.7670	Allergen			ANAILGVSLAAAKAA	108–122	1.3127	No
		VLNGGSHAGGALAFQ	155–169	0.3967	No			LNGGSHAGGALAFQE	155–169	0.6505	Allergen
		LGANAILGVSLAAAN	107–121	1.9257	No			QNVLNGGSHAGGALA	152–166	0.6131	Allergen
		GANAILGVSLAAANA	108–122	1.7898	No			NGGSHAGGALAFQEF	156–170	0.6872	No
		LNGGSHAGGALAFQE	156–170	0.6505	Allergen			LGANAILGVSLAAAK	106–120	2.1683	No
		AANAAAAAQGIPLYK	119–133	0.6124	No			GANAILGVSLAAAKA	107–121	1.9809	No
		QNVLNGGSHAGGALA	153–167	0.6131	Allergen			FQNVLNGGSHAGGAL	151–165	1.9809	No
		NGGSHAGGALAFQEF	157–171	0.6872	No			GGSHAGGALAFQEFM	157–171	1.2248	No
		FQNVLNGGSHAGGAL	152–166	0.1721	No			KLGANAILGVSLAAA	105–119	1.7237	No
		GGSHAGGALAFQEFM	158–172	1.2248	No			AAAKAAAAEKGVSLY	117–131	0.8440	No
		KLGANAILGVSLAAA	106–120	1.7237	No			PFQNVLNGGSHAGGA	150–164	0.3371	No
		ANAAAAAQGIPLYKH	120–134	0.7353	Allergen			GSHAGGALAFQEFMI	158–172	1.3377	No
		PFQNVLNGGSHAGGA	151–165	0.3371	No			SKLGANAILGVSLAA	104–118	1.7264	No
		GSHAGGALAFQEFMI	159–173	1.3377	No			LFRAIVPSGASTGVH	30–44	-0.6248	No
		ANDSYAAGWGVMVSH	363–377	2.3479	Allergen			FRAIVPSGASTGVHE	31–45	-0.1450	No
		NDSYAAGWGVMVSHR	364–378	2.0051	Allergen			AAKAAAAEKGVSLYK	118–132	0.5945	No
		AANDSYAAGWGVMVS	362–376	2.5202	Allergen			LRSGQIKTGAPARSE	393–407	-0.8320	Allergen
		SKLGANAILGVSLAA	105–119	1.7264	No			RSGQIKTGAPARSER	394–408	-0.2652	Allergen
		DSYAAGWGVMVSHRS	365–379	1.9971	No			RAIVPSGASTGVHEA	32–46	0.1543	No
		LRSGQIKTGAPARSE	394–408	-0.8320	Allergen			KSKLGANAILGVSLA	103–117	1.8868	Allergen
		RSGQIKTGAPARSER	395–409	-0.2652	Allergen			AKAAAAEKGVSLYKH	119–133	0.6301	No
		LFRSIVPSGASTGVH	31–45	-0.7808	Allergen			SGQIKTGAPARSERL	395–409	0.4913	No
		FRSIVPSGASTGVHE	32–46	-0.3224	Allergen			NKSKLGANAILGVSL	102–116	1.8168	No
		KSKLGANAILGVSLA	104–118	1.8868	Allergen			GQIKTGAPARSERLA	396–410	0.3947	Allergen
		SGQIKTGAPARSERL	396–410	0.4913	No			AIVPSGASTGVHEAL	33–47	0.4391	Allergen
		NKSKLGANAILGVSL	103–117	1.8168	No			LDLIVAAIEKAGYTG	226–240	-0.1497	No
		GQIKTGAPARSERLA	397–411	0.3947	Allergen			GLRSGQIKTGAPARS	392–406	-0.5697	Allergen
		RSIVPSGASTGVHEA	33–47	0.0413	No						
		SIVPSGASTGVHEAL	34–48	0.3859	Allergen						
		GLRSGQIKTGAPARS	393–407	-0.5697	Allergen						
3	HLA-DRB1*01:01	KGLFRSIVPSGASTG	29–43	-1.4352	No	3	HLA-DRB1*01:01	DKGLFRAIVPSGAST	27–41	-1.6360	No
		TTDKGLFRSIVPSGA	26–40	-1.5357	Allergen			KGLFRAIVPSGASTG	28–42	-1.3664	No
		TDKGLFRSIVPSGAS	27–41	-1.8364	Allergen			GLFRAIVPSGASTGV	29–43	-0.6347	No
		DKGLFRSIVPSGAST	28–42	-1.7048	Allergen			ITDKGLFRAIVPSGA	25–39	-2.4716	No
		GLFRSIVPSGASTGV	30–44	-0.7221	No			TDKGLFRAIVPSGAS	26–40	-1.7676	No
		ILGVSLAAANAAAAA	112–126	1.1319	No			AILGVSLAAAKAAAA	110–124	1.1800	No
		AILGVSLAAANAAAA	111–125	0.9584	No			ILGVSLAAAKAAAAE	111–125	1.4324	Allergen
		LGVSLAAANAAAAAQ	113–127	1.4840	No			LGVSLAAAKAAAAEK	112–126	1.7272	Allergen
		NAILGVSLAAANAAA	110–124	1.1003	No			GVSLAAAKAAAAEKG	113–127	0.9995	Allergen
		ANAILGVSLAAANAA	109–123	1.1338	No			VSLAAAKAAAAEKGV	114–128	0.6287	Allergen
		GVSLAAANAAAAAQG	114–128	1.1569	Allergen			NAILGVSLAAAKAAA	109–123	1.2659	No
		SEVYHNLKSLTKKKY	189–203	-0.0101	No			ANAILGVSLAAAKAA	108–122	1.3127	No
		GSEVYHNLKSLTKKK	188–202	-0.0897	No			SEVYHNLKSLTKKKY	188–202	-0.0101	No
		EVYHNLKSLTKKKYG	190–204	0.6927	No			GSEVYHNLKSLTKKK	187–201	-0.0897	No
		IGSEVYHNLKSLTKK	187–201	0.0822	No			EVYHNLKSLTKKKYG	189–203	0.6927	No
		RIGSEVYHNLKSLTK	186–200	-0.2144	No			YGSEVYHNLKSLTKK	186–200	-0.0341	No
		VSLAAANAAAAAQGI	115–129	0.7498	No			GANAILGVSLAAAKA	107–121	1.9809	No
		LFRSIVPSGASTGVH	31–45	-0.7808	No			RYGSEVYHNLKSLTK	185–199	-0.2436	No
		FRSIVPSGASTGVHE	32–46	-0.3224	Allergen			LFRAIVPSGASTGVH	30–44	-0.6248	No
		ETEDTFIADLSVGLR	381–395	1.1540	Allergen			FRAIVPSGASTGVHE	31–45	-0.1450	No
		TEDTFIADLSVGLRS	382–396	0.9944	Allergen						
		GETEDTFIADLSVGL	380–394	0.9944	No						
		EDTFIADLSVGLRSG	383–397	0.1815	Allergen						
		DTFIADLSVGLRSGQ	384–398	0.4133	Allergen						
		GANAILGVSLAAANA	108–122	1.7898	No						
		IPLYKHIANISNAKK	129–143	0.9816	Allergen						
		GIPLYKHIANISNAK	128–142	0.7118	No						
		QGIPLYKHIANISNA	127–141	-0.0059	Allergen						
		DSYAAGWGVMVSHRS	365–379	1.9971	No						
		AQGIPLYKHIANISN	126–140	0.3429	Allergen						
		PLYKHIANISNAKKG	130–144	0.3329	Allergen						
		LAFQEFMIAPTGVST	166–180	0.0115	Allergen						
4	HLA-DRB1*04:04	IPLYKHIANISNAKK	129–143	0.9816	Allergen	4	HLA-DRB1*04:04				
		AQGIPLYKHIANISN	126–140	0.3429	Allergen						
		QGIPLYKHIANISNA	127–141	-0.0059	Allergen						
		GIPLYKHIANISNAK	128–142	0.7118	No						
		PLYKHIANISNAKKG	130–144	0.3329	Allergen						
5	HLA-DRB1*04:05	IPLYKHIANISNAKK	129–143	0.9816	Allergen	5	HLA-DRB1*04:05				
		AQGIPLYKHIANISN	126–140	0.3429	Allergen						
		QGIPLYKHIANISNA	127–141	-0.0059	Allergen						
		GIPLYKHIANISNAK	128–142	0.7118	No						
		PLYKHIANISNAKKG	130–144	0.3329	Allergen						
6	HLA-DRB1*07:01	GDDLTVTNPTRIKTA	323–337	2.6314	Allergen	6	HLA-DRB1*07:01	QIVGDDLTVTNPIRI	319–333	3.0425	Allergen
		QIVGDDLTVTNPTRI	320–334	2.7496	Allergen			IVGDDLTVTNPIRIK	320–334	2.9859	Allergen
		IVGDDLTVTNPTRIK	321–335	2.7815	Allergen			VGDDLTVTNPIRIKT	321–335	2.6573	No
		VGDDLTVTNPTRIKT	322–336	2.6563	No			GDDLTVTNPIRIKTA	322–336	2.6008	Allergen
		DDLTVTNPTRIKTAI	324–338	1.2677	No			DDLTVTNPIRIKTAI	323–337	1.2371	No
								MAVTKIHARSVYDSR	1–15	0.2231	No
7	HLA-DRB1*09:01	AANDSYAAGWGVMVS	362–376	2.5202	Allergen	7	HLA-DRB1*09:01	ITDKGLFRAIVPSGA	25–39	-2.4716	No
		ANDSYAAGWGVMVSH	363–377	2.3479	Allergen			TDKGLFRAIVPSGAS	26–40	-1.7676	No
		NDSYAAGWGVMVSHR	364–378	2.0051	Allergen			DKGLFRAIVPSGAST	27–41	-1.6360	No
		DSYAAGWGVMVSHRS	365–379	1.9971	No			KGLFRAIVPSGASTG	28–42	-1.3664	No
		QAANDSYAAGWGVMV	361–375	1.9931	Allergen			GLFRAIVPSGASTGV	29–43	-0.6347	No
		TDKGLFRSIVPSGAS	27–41	-1.8364	Allergen			LGVSLAAAKAAAAEK	112–126	1.7272	Allergen
		DKGLFRSIVPSGAST	28–42	-1.7048	Allergen			AILGVSLAAAKAAAA	110–124	1.1800	No
		KGLFRSIVPSGASTG	29–43	-1.4352	No			ILGVSLAAAKAAAAE	111–125	1.4324	Allergen
		TTDKGLFRSIVPSGA	26–40	-1.5357	Allergen			ESWSKFYPRVVDKIQ	305–319	-0.6677	No
		GLFRSIVPSGASTGV	30–44	-0.7221	No			GVSLAAAKAAAAEKG	113–127	0.9995	Allergen
								WESWSKFYPRVVDKI	304–318	-0.6064	Allergen
								VSLAAAKAAAAEKGV	114–128	0.6287	Allergen
								DDWESWSKFYPRVVD	302–316	0.2325	Allergen
								DWESWSKFYPRVVDK	303–317	0.0020	Allergen
								EDDWESWSKFYPRVV	301–315	-0.1906	No
8	HLA-DRB1*11:01	SEVYHNLKSLTKKKY	189–203	-0.0101	No	8	HLA-DRB1*11:01	SEVYHNLKSLTKKKY	188–202	-0.0101	No
		EVYHNLKSLTKKKYG	190–204	0.6927	No			EVYHNLKSLTKKKYG	189–203	0.6927	No
		VYHNLKSLTKKKYGQ	191–205	0.7173	No			VYHNLKSLTKKKYGL	190–204	0.5890	No
		YHNLKSLTKKKYGQS	192–206	1.3583	No			YHNLKSLTKKKYGLS	191–205	1.4473	No
		HNLKSLTKKKYGQSA	193–207	1.5922	No			HNLKSLTKKKYGLSA	192–206	1.8209	No
		GSEVYHNLKSLTKKK	188–202	-0.0897	No			GSEVYHNLKSLTKKK	187–201	-0.0897	No
		IGSEVYHNLKSLTKK	187–201	0.0822	No			YGSEVYHNLKSLTKK	186–200	-0.0341	No
		RIGSEVYHNLKSLTK	186–200	-0.2144	No			RYGSEVYHNLKSLTK	185–199	-0.2436	No
9	HLA-DRB3*01:01	GETEDTFIADLSVGL	380–394	1.5303	No	9	HLA-DRB3*01:01	QAAKDSYAADWGVMV	360–374	1.8143	No
		ETEDTFIADLSVGLR	381–395	1.1540	Allergen			AAKDSYAADWGVMVS	361–375	2.2518	No
		TEDTFIADLSVGLRS	382–396	0.9944	Allergen			AKDSYAADWGVMVSH	362–376	2.1024	Allergen
		EDTFIADLSVGLRSG	383–397	0.1815	Allergen			KDSYAADWGVMVSHR	363–377	1.7315	Allergen
		DTFIADLSVGLRSGQ	384–398	0.4133	Allergen			DSYAADWGVMVSHRS	364–378	1.6410	Allergen
10	HLA-DRB5*01:01	GSEVYHNLKSLTKKK	188–202	-0.0897	No	10	HLA-DRB5*01:01	GSEVYHNLKSLTKKK	187–201	-0.0897	No
		SEVYHNLKSLTKKKY	189–203	-0.0101	No			SEVYHNLKSLTKKKY	188–202	-0.0101	No
		EVYHNLKSLTKKKYG	190–204	0.6927	No			EVYHNLKSLTKKKYG	189–203	0.6927	No
		RIGSEVYHNLKSLTK	186–200	-0.2144	No			RYGSEVYHNLKSLTK	185–199	-0.2436	No
		IGSEVYHNLKSLTKK	187–201	0.0822	No			YGSEVYHNLKSLTKK	186–200	-0.0341	No
		VYHNLKSLTKKKYGQ	191–205	0.7173	No			VYHNLKSLTKKKYGL	190–204	0.5890	No
		EVDFTTDKGLFRSIV	22–36	0.4425	No			YHNLKSLTKKKYGLS	191–205	1.4473	No
		VDFTTDKGLFRSIVP	23–37	-0.0369	No			AILGVSLAAAKAAAA	110–124	1.1800	No
		YHNLKSLTKKKYGQS	192–206	1.3583	No			ANAILGVSLAAAKAA	108–122	1.3127	No
		VEVDFTTDKGLFRSI	21–35	1.0234	No			NAILGVSLAAAKAAA	109–123	1.2659	No
		PTVEVDFTTDKGLFR	19–33	1.3049	No						
		TVEVDFTTDKGLFRS	20–34	1.1140	No						
11	HLA-DPA1*01:03/DPB1*02:01					11	HLA-DPA1*01:03/DPB1*02:01	SHAGGALAFQEFMIV	159–173	0.7509	Allergen
								HAGGALAFQEFMIVP	160–174	0.3523	Allergen
								AGGALAFQEFMIVPS	161–175	0.4696	Allergen
								GGALAFQEFMIVPSG	162–176	-0.0871	No
								GALAFQEFMIVPSGA	163–177	-0.8395	No
12	HLA-DRB1*03:01					12	HLA-DRB1*03:01	EVDLITDKGLFRAIV	21–35	-0.8399	No
								VDLITDKGLFRAIVP	22–36	-1.3038	No
13	HLA-DRB1*11:01					13	HLA-DRB1*11:01	SEVYHNLKSLTKKKY	188–202	-0.0101	No
								EVYHNLKSLTKKKYG	189–203	0.6927	No
								VYHNLKSLTKKKYGL	190–204	0.5890	No
								YHNLKSLTKKKYGLS	191–205	1.4473	No
								HNLKSLTKKKYGLSA	192–206	1.8209	No
								GSEVYHNLKSLTKKK	187–201	-0.0897	No
								YGSEVYHNLKSLTKK	186–200	-0.0341	No
								RYGSEVYHNLKSLTK	185–199	-0.2436	No

*Antigenicity and Allergenicity values predicted by VaxiJen and AllerTOP servers, respectively.

## Discussion

This research work focuses on the molecular cloning, protein expression, followed by purification of recombinant Enolase proteins from both *C. albicans* and *C. auris*, along with *in silico* prediction and identification of B-cell and T-cell epitopes. Our study contributes to the ongoing efforts to develop effective vaccines for preventing and controlling infections caused by the multidrug-resistant *C. auris* infections. Further, our results may also help in developing improved diagnostic strategies for *C. auris* infections. *C. auris* has emerged as a significant concern due to its ability to cause systemic infections, its propensity for spreading within healthcare settings and is associated with high morbidity and mortality across the globe (Chowdhary et al., 2023). Since its initial discovery in Japan in 2009, *C. auris* has rapidly spread across the globe, causing outbreaks in healthcare facilities in various countries (Spivak and Hanson, 2018). It exhibits significant genetic diversity, with six distinct clades namely South Asian (I), East Asian (II), African (III), South American (IV), Iran (V) and Singapore (VI) identified across different geographic regions (Narayanan et al., 2022; Spruijtenburg et al., 2022; Suphavilai et al., 2023). Unlike other *Candida* species, *C. auris* is resistant to multiple classes of antifungal treatments including azoles, echinocandins, and amphotericin B, making it difficult to treat. Identifying *C. auris* infections can be challenging, as it may be misidentified as other *Candida* species using conventional laboratory methods, lead to delayed diagnosis and inappropriate treatment, potentially exacerbating the spread of the infection. Despite advancements in understanding host defense mechanisms, invasive infections caused by *C. auris* remain poorly understood. Improved understanding of host immune responses during *C. auris* infections has important clinical implications, including the development of immunotherapeutic associated with systemic candidiasis (Pechacek and Lionakis, 2023).

Currently, there are no approved immunotherapeutic strategies/vaccines specifically designed for the treatment of systemic candidiasis, including those caused by multidrug-resistant *C. auris*. Of note, host-directed therapy and vaccine development (NDV-3) for *C. auris* mediated systemic candidiasis is still in progress (Singh et al., 2019). Akhtar et al. used bioinformatics tools and databases to identify potential antigenic epitopes from agglutinin-like sequence-3 (Als3) proteins expressed by *C. auris* (Akhtar et al., 2021). In addition to Als3, the Enolase protein exhibits significant conservation across various *Candida* species, positioning it as a prominent vaccine candidate for systemic candidiasis. This recognition makes it a promising focus for the development of broad-spectrum vaccines (Shukla et al., 2021b). Xin et al. conducted a study demonstrating the creation of synthetic glycopeptide vaccines that combine beta-mannan with peptide epitopes derived from the Enolase protein of *C. albicans*. These vaccines were shown to provide protection against systemic candidiasis in a mouse model (Xin et al., 2008). Besides being considered a potential vaccine candidate, Enolase protein also has a role in diagnosing systemic candidiasis infections (He et al., 2015). The immunogenic ability of Enolase is well documented and several studies have investigated the use of recombinant Enolase protein from *C. albicans* for diagnostic potential (Laín et al., 2007; Pitarch et al., 2014). Detection of Enolase-specific antibodies has been used in the diagnosis of systemic candidiasis in multiple reports (van Deventer et al., 1994; Li et al., 2013). As such, recombinant Enolase protein belonging to *Candida* species exhibits both diagnostic and therapeutic potential against systemic *Candida* infections.

In this study, we successfully cloned and expressed recombinant Enolase proteins from South Asian strain of *C. auris* (clade I CBS 470280) and *C. albicans* reference strain (SC5314) in *E. coli*. The recombinant Enolase protein from *C. albicans* has previously been cloned and expressed using prokaryotic (Mason et al., 1993) and eukaryotic expression systems (Franklyn and Warmington, 1993) and characterized (Sandini et al., 1999). Another study has characterized the sequence and structural features of the *C. krusei* Enolase protein using *in silico* methods (Gandhi et al., 2008). We performed structural and immunoinformatics analysis (involving *in silico* B-cell and T-cell epitope analysis) for comparing the two proteins. *In silico* immunoinformatics analysis indicated that the B-cell and T-cell epitopes were comparable in both the Enolase proteins from *C. albicans* and *C. auris*. We used BepiPred and DiscoTope servers available at IEDB to predict the linear and discontinuous B-cell epitopes, respectively. The antigenicity and allergenicity analyses for the identified epitopes were performed using VaxiJen and AllerTOP servers, respectively (Mir et al., 2022). Enolase protein from *C. auris* was observed to have less discontinuous epitopes than the Enolase protein of *C. albicans* in this study. It can be explained by the fact that their precise number and locations cannot be determined due to the lack of experimental structural data of this protein. We used modeled tertiary structure of the Enolase protein from *C. auris* using the Swiss Model server with an 82% template similarity with *C. albicans* Enolase protein, offering valuable insights into potential epitope distribution. However, the exact disposition of discontinuous epitopes remains uncertain without experimental validation. We used NetMHCpan 4.1 EL and NETMHCIIpan 4.1 EL prediction tools available at IEDB for predicting the Enolase CD8 and CD4 T-cell epitopes, respectively. While both the total number and antigenic CD8 and CD4 T-cell epitopes were comparable between Eno-albicans and Eno-auris proteins, the Enolase protein from *C. auris* exhibited lesser number of allergenic epitopes compared to the protein from *C. albicans*. The crystal structure of Enolase protein from *C. albicans* is available and provides valuable insights into its pathogenesis, virulence, and potential as a therapeutic vaccine candidate (Li et al., 2022). The study by Langenhorst et al. highlighted the immunostimulatory and therapeutic potential of Enolase protein from *C. albicans* on human and mouse immune cells (Langenhorst et al., 2023). Another study demonstrated direct modulation of T cell responses against *C. albicans* infections (Daud et al., 2023). Our results suggest that Enolase epitopes augers well for designing an improved anti-*Candida* vaccine. However, a multi-epitope vaccine comprising additional epitopes from various other immunogenic proteins would enhance vaccine efficacy. Along with Enolase, we recommend specific proteins such as secreted aspartyl proteinase (Sap2), agglutinin like sequence gene (Als3), heat shock protein (Hsp90), hyphally-regulated protein (Hyr1), hyphal wall protein (Hwp1), phospholipase (PLB), pyruvate kinase (Pk), fructose bisphosphate aldolase (Fba1), superoxide dismutase gene (Sod5), malate dehydrogenase (Mdh1), etc. and their epitopes for inclusion in such a combination approach, based on their known immunogenicity and relevance to *Candida* infection (Shukla et al., 2021b).

Enolase protein holds promise as both a diagnostic marker and a therapeutic target against multidrug resistance *Candida* infections. Further research into the development of Enolase protein based diagnostic assays, and multi-epitope vaccines in preclinical and clinical studies may contribute to improved strategies for diagnosing, preventing, and treating *Candida* infections in clinical settings. Overall, this study lays the groundwork for further investigation into the immunogenicity and protective efficacy of Enolase-based vaccines against *Candida* infections. These findings underscore the potential of Enolase as a versatile vaccine candidate for combating *Candida*-associated diseases and highlight the importance of molecular approaches in vaccine development against fungal pathogens. The emergence of Covid associated fungal infections and global warming increasing *C. auris* infections has provided momentum to fungal vaccine research. The availability of an improved multivalent and/or multi-epitope vaccine for pan-fungal infections is on the horizon, within next 10 years, hopefully.

## Data availability statement

The original contributions presented in the study are included in the article/supplementary material. Further inquiries can be directed to the corresponding author.

## Author contributions

MS: Data curation, Formal analysis, Investigation, Methodology, Visualization, Writing – original draft. RS: Data curation, Formal analysis, Investigation, Visualization, Writing – review & editing. PC: Data curation, Investigation, Visualization, Writing – review & editing. SR: Conceptualization, Formal analysis, Methodology, Project administration, Resources, Supervision, Validation, Writing – review & editing.
